# Severe forms of influenza infections admitted in intensive care units: Analysis of mortality factors

**DOI:** 10.1111/irv.13168

**Published:** 2023-07-21

**Authors:** Victor Verdier, François Lilienthal, Arnaud Desvergez, Virgile Gazaille, Arnaud Winer, Fabrice Paganin

**Affiliations:** ^1^ Service de Réanimation CHU Réunion Saint‐Denis France; ^2^ Taniken LLC Tampa Florida USA; ^3^ Cabinet de Pneumologie Le Port France; ^4^ Service de Pneumologie CHU Réunion Saint‐Denis France

**Keywords:** ARDS, influenza, intensive care, mortality, prognosis factors

## Abstract

**Background:**

The severe forms of influenza infection requiring intensive care unit (ICU) admission remain a medical challenge due to its high mortality. New H1N1 strains were hypothesized to increase mortality. The studies below represent a large series focusing on ICU‐admitted influenza patients over the last decade with an emphasis on factors related to death.

**Methods:**

A retrospective study of patients admitted in ICU for influenza infection over the 2010–2019 period in Réunion Island (a French overseas territory) was conducted. Demographic data, underlying conditions, and therapeutic management were recorded. A univariate analysis was performed to assess factors related to ICU mortality.

**Results:**

Three hundred and fifty adult patients were analyzed. Overall mortality was 25.1%. Factors related to higher mortality were found to be patient age >65, cancer history, need for intubation, early intubation within 48 h after admission, invasive mechanical ventilation (MV), acute respiratory distress syndrome (ARDS), vaso‐support drugs, extracorporal oxygenation by membrane (ECMO), dialysis, bacterial coinfection, leucopenia, anemia, and thrombopenia. History of asthma and oseltamivir therapy were correlated with a lower mortality. H1N1 did not impact mortality.

**Conclusion:**

Patient's underlying conditions influence hospital admission and secondary ICU admission but were not found to impact ICU mortality except in patients age >65, history of cancer, and bacterial coinfections. Pulmonary involvement was often present, required MV, and often evolved toward ARDS. ICU mortality was strongly related to ARDS severity. We recommend rapid ICU admission of patients with influenza‐related pneumonia, management of bacterial coinfection, and early administration of oseltamivir.

## 1 BACKGROUND

In 2009, a new H1N1 influenza virus emerged, causing the first global flu pandemic in 40 years.^1^, ^2^ A rapidly increasing incidence of severe forms of influenza infections created concern among the medical community and was responsible for an increase in intensive care unit (ICU) admissions.^3^, ^4^

Réunion Island is a French tropical overseas territory located in the south hemisphere in the Indian Ocean. It benefits from a survey system to monitor influenza cases. Influenza has a seasonal incidence that is less pronounced than in temperate countries such as mainland France.^5^, ^6^ The burden of influenza's annual outbreak in the general population is difficult to assess. However, severe forms of influenza admitted in ICU should benefit from extensive data collection.

In this retrospective analysis, the severe cases of influenza infections admitted to the ICU in Réunion Island from 2010 to2019 and the factors related to poor outcomes and death were described and analyzed.

## 2 METHODS

### 2.1 Patients

Patients were admitted to the ICU of the two tertiary teaching hospitals in Réunion Island (St‐Denis and St‐Pierre). Reunion Island has a population of about 850, 000 people. The medical system and accessibility to medical care is not different to that of mainland France.

The data of 379 patients admitted in ICU between January 2010 and December 2019 were retrospectively analyzed. Twenty‐seven cases were excluded because of age <18 years. Two other cases were excluded: one for false diagnosis and one for the lack of a definite diagnosis. A total of 350 adult patients met with definite diagnosis assessed by a positive real‐time polymerase chain reaction (RT‐PCR) nasopharyngeal swab or a tracheal aspirate were included in the analysis.

ICU admission demographic data, underlying conditions such as diabetes mellitus, asthma, chronic obstructive pulmonary disease (COPD), obstructive sleep apnea (OSA), chronic renal failure, hypertension, coronary diseases, liver diseases, pregnancy, obesity, prior illness or treatment (history of cancer, immunosuppressive therapy), alcohol abuse and smoking habits, standard biological variables, and information on vaccine status within the current year were collected.

Simplified acute physiologic score II (SAPS II) was calculated at admission.

The need for invasive mechanical ventilation (MV), administration of vasopressor drugs within the first 48 h after admission, dialysis requirement in the case of acute renal failure, length of MV, and intubation were recorded during ICU stay. The use of antiviral for treatment of influenza before and during ICU stay was recorded.

Acute respiratory distress syndrome (ARDS) was defined according to guidelines.^7^ The use of extracorporal oxygenation by membrane (ECMO) was performed as needed.

Concomitant bacterial infections were defined if a significant predominant strain was found with a cut point >10^4^/mL in bronchoalveolar lavage (BAL) or >10^3^/mL in protected distal aspiration.

### 2.2 Statistical analysis

Prognostic factors for mortality were analyzed using the chi‐squared test with Fisher's exact test correction, if needed. A *p* value < 0.05 was considered statistically significant.

The relative risk (RR) for outcome was defined according to the following variables: age (>65 or <65 years), obesity (BMI > 30 or <30), COPD (Y/N), asthma (Y/N), OSA (Y/N), diabetes mellitus (Y/N), hypertension (Y/N), coronary disease (Y/N), liver cirrhosis (Y/N), chronic renal failure with dialysis (Y/N), alcohol abuse (>80 g/day or <80 g/day), tobacco (active or past Y/N), pregnancy for females under 41 years of age (Y/N), history of cancer (Y/N), immunosuppressive drugs including oral steroids >10 mg per day (Y/N), SPAS II score (>40 or <40), intubation during stay (Y/N), need of MV within first 48 h after admission (Y/N), MV during stay (>10 days or <10 days), vasopressor drugs (Y/N), ARDS (Y/N), ECMO (Y/N), H1N1 strains versus others (Y/N), concomitant bacterial infections (Y/N), pneumococcal strains versus all (Y/N), staphylococcus strains versus all (Y/N), oseltamivir administration (Y/N), polynuclear cells (<2.5 G/L or >2.5 G/L), hemoglobin (<8 g/dL or >8 g/dL), platelets (<50 G/L or >50 G/L), and C‐reactive protein (>100 mg/L or <100 mg/L).

The study received approval from the institutional review board (IRB) and the Comité National de l'informatique et des Libertés (CNIL).

## 3 RESULTS

Among 350 patients, 205 (58.5%) were male. The mean of age was 58.9 +/− 15.2 years (19–88). The patient's characteristics, underlying conditions, and prior illnesses are presented in Table 1. The major underlying conditions were hypertension (51%), smoking (48.6%), diabetes mellitus (37.4%), COPD (23%), and coronary diseases (20%).

**TABLE 1:** Patient's characteristics, underlying conditions, and prior illnesses.

	*N* (%) or mean +/− SD	Number of patients with data available (%)
Sex (M/F)	205 (58.5%)/145 (41.5%)	350 (100%)
Mean age (years)	58.9 +/− 15.2	350 (100%)
Duration of ICU stay (day)	12.3 +/− 14.3	350 (100%)
Obesity (BMI > 30)	77 (18.3%)	198 (56.6%)
Mean BMI	28.2 +/− 7.8	198 (56.6%)
Smoking habit	170 (48.6%)	350 (100%)
COPD	75 (21.7%)	345 (98.6%)
OSA	46 (13%)	348 (99.4%)
Asthma	63 (18.1%)	349 (99.7%)
Diabetes mellitus	130 (37.4%)	348 (99.4%)
Hypertension	180 (51.4%)	350 (100%)
Coronary disease	69 (20%)	343 (98%)
Alcohol abuse	75 (21.4%)	350 (100%)
Liver cirrhosis	13 (3.7%)	349 (99.7%)
Chronic dialysis	13 (3.7%)	350 (100%)
History of cancer	40 (11.5%)	349 (99.7%)
Ongoing immunosuppression	16 (4.6%)	348 (99.4%)
Pregnancy (females age <41)	13 (48%)	27 (100%)

The mean SAPS II score was 43.8 +/− 20.3. The mean duration of ICU stay was 12.3 +/− 14.3 days (1–122).

A total of 204 (58%) patients required MV, with 185 of them intubated within the first 48 h of ICU stay. The severity of patients was further documented by the use of vaso‐drug support (51.5%), dialysis (19.1%), and ECMO (7.7%) and by the onset of ARDS (40.2%).

ARDS was recorded in 134 patients according to Berlin guidelines. The ARDS severity grade for 107 patients was recorded. Grade III (ratio of PaO2/FIO2 < 100) for 86 (80.4%) and grade II (ratio of PaO2/FIO2 < 200) for 17 (15.9%) patients, respectively, were found.

H1N1 strains were found in 153 (43.7%) cases and were the predominant influenza type followed by B 68 (19.4%), H3Nx 66 (18.9%), and non‐defined A strains 63 (18%).

Bacterial coinfections were observed in 116 patients and were correlated with mortality (*p* < 0.04). Data revealed pneumococcal stains in 28 (24.1%) cases and *Staphylococcus aureus* in 40 (34.5%) cases. Staphylococcus and streptococcus coinfection was not correlated with mortality. Other agents are listed as follows: wild strains *Pseudomonas aeruginosa* 9 (7.8%), *Streptococcus* species 7 (6%), *Klebsiella pneumoniae* 6 (5.2%), *Moraxella catarrhalis* 4 (3.4%), *Enterobacter* 3 (2.6%), *Haemophilus influenzae* 3 (2.6%), *Legionella pneumophila* 3 (2.6%), and others 13 (11.2%).

Oseltamivir was the unique antiviral drug used in this study and was administrated in 229 (66.4%) inpatients, according to hospital's attending physician's judgment. Only 16/96 (16.6%) patients were vaccinated against influenza with no impact on mortality (*p* < 0.29).

The overall mortality was 25.1% (88/350).

All conditions and care are presented in Table 2.

**TABLE 2:** Clinical conditions and care in ICU.

	*N* (%) or mean +/− SD	Number of patients with data available (%)
SAPS II score	43.8 +/− 20.3	347 (99.1%)
Noninvasive ventilation only	81 (23.1%)	350 (100%)
Intubation and MV	204 (58%)	350 (100%)
Intubation duration (days)	12.3 +/− 14.3	350 (100%)
Intubation within 48 h	185 (52.9%)	350 (100%)
ARDS	134 (40.2%)	333 (95.1%)
Vaso‐support drugs within 48 h	175 (51.5%)	340 (97.1%)
ECMO	27 (7.7%)	350 (100%)
ECMO duration (days)	19.1 +/− 24.0	
Dialysis requirement	64 (19.1%)	335 (95.7%)
Oseltamivir therapy	229 (66.4%)	345 (98.6%)
H1N1	153 (43.7%)	350 (100%)
Vaccination status	16 (16.6%)	96 (27.4%)
Concomitant bacterial infection	116 (35.8%)	324 (92.6%)
Pneumococcus *Staphylococcus aureus*	28 (8.6%) 40 (12.3%)	324 (100%)
Leucocytes G/L	9.399 +/− 6.202	347 (99.1%)
Hemoglobin g/dL	12.5 +/− 2.5	347 (99.1%)
Platelets G/L	179 +/− 79	347 (99.1%)
Creatinine μmol/L	167 +/− 173	347 (99.1%)
ASAT UI/L	138 +/− 336	333 (95.1%)
ALAT UI/L	83 +/− 232	333 (95.1%)
CRP mg/L	155 +/− 141	202 (57.7%)
Overall mortality	88 (25.1%)	350 (100%)

The univariate analysis recorded variables related to death are listed in Table 3. The univariate analysis identified the following variables as potential prognostic risk factors of death: age over 65, history of cancer, SAPS II score over 40, intubation during stay and within the first 48 h after admission, vaso‐drug support, ARDS, requirement of ECMO, dialysis, bacterial coinfections, leucocytes <2.5 G/L, hemoglobin <8 g/dL, and platelets <50 G/L.

**TABLE 3:** Univariate analysis for clinical factors, therapeutic issues, and laboratory findings in surviving versus non‐surviving patients in ICU.

	Non‐survivors *N* = 88 (%)	Survivors *N* = 262 (%)	Relative risk	95% CI	*P* value
Age >65 years	46/88 (52.3%)	88/262 (33.6%)	1.55	1.18–2.0	0.002
Male/female	51/37	154/108	0.9	0.79–1.19	0.9
Obesity BMI > 30	18/53 (34%)	59/145 (40.6%)	0.8	0.49–1.3	0.41
Smoking habits	38/88 (43.2%)	132/262 (50.5%)	0.8	0.55–1.15	0.26
COPD	18/86 (20.9%)	57/259 (22%)	0.95	0.59–1.46	0.88
OSA	8/87 (9.2%)	38/261 (14.5%)	0.66	0.33–1.21	0.27
Asthma	6/87 (6.9%)	57/262 (21.7%)	0.33	0.15–0.69	0.0012
Diabetes mellitus	32/88 (36.4%)	98/260 (37.7%)	0.95	0.65–1.38	0.89
Hypertension	38/88 (42.7%)	142/262 (54%)	0.71	0.49–1.02	0.07
Coronary disease	22/84 (26.1%)	47/259 (18.2%)	1.4	0.92–2.1	0.11
Alcohol abuse	16/88 (18.2%)	59/262 (22.5%)	0.81	0.49–1.28	0.45
Liver cirrhosis	5/87 (5.75%)	8/262 (3.1%)	1.56	0.71–2.76	0.32
Chronic dialysis	5/88 (5.7%)	8/262 (3%)	1.55	0.7–2.72	0.32
History of cancer	17/88 (19.3%)	23/261 (8.8%)	1.85	1.87–2.7	0.011
Immunosuppression	6/88 (6.8%)	10/260 (3.85%)	1.51	0.73–2.6	0.24
Pregnancy (females <41 years)	3/6 (50%)	10/21 (47.6%)	1.07	0.28–4	1
Duration of stay >10 days	32/88 (36.4%)	101/262 (38.5%)	0.93	0.63–1.35	0.79
SAPS II score >40	71/84 (77.2%)	101/261 (38.7%)	4.81	2.91–8.1	< 0.001
Intubation within 48 h	68/88 (77.2%)	117/262 (44.7%)	3	1.95–4.78	< 0.0001
Intubation during stay	74/88 (84.1%)	130/262 (49.6%)	3.78	2.66–6.44	0.0001
MV > 14 days	25/74 (33.8%)	46/129 (36.6%)	0.94	0.63–1.37	0.87
Vaso‐support drugs within 48 h	66/82 (80.5%)	109/258 (42.3%)	3.89	2.38–6.44	< 0.0001
ARDS	61/82 (74.4%)	73/251 (29.8%)	4.31	2.79–6.73	< 0.0001
ECMO	21/88 (23.8%)	6/262 (2.3%)	3.75	2.68–4.92	< 0.0001
Dialysis (except previous chronic dialysis)	39/81 (48.2%)	25/254 (9.8%)	3.93	2.78–5.5	< 0.0001
Vaccination	1/55 (6.7%)	15/65 (23.1%)	0.29	0.0064–2.2	0.29
Oseltamivir Administration	48/85 (56.5%)	181/260 (69.6%)	0.65	0.45–0.95	0.03
H1N1	38/88 (43.2%)	115/262 (43.9%)	0.97	0.67–1.4	0.99
Bacterial concomitant infection	38/84 (45.2%)	78/240 (32.5%)	1.43	1.2–2.05	0.04
Pneumococcus vs. non‐concomitant infection	7/53 (13.2%)	21/183 (11.5%)	1.17	0.4–3.1	0.8
*Staphylococcus aureus* vs. non‐concomitant infection	13/53 (24.5%)	27/189 (14.2%)	1.67	0.7–3.7	0.16
Leucocytes <2.5 G/L	12/88 (13.6%)	11/259 (4.3%)	2.24	1.35–3.24	0.005
Hemoglobin <8 g/dL	8/88 (9.1%)	8/259 (3.1%)	2.06	1.13–3.17	0.03
Platelet <50 G/L	5/88 (5.6%)	3/259 (1.2%)	2.55	1.22–3.8	0.02
CRP >100 mg/L	26/39 (66.7%)	82/163 (50.3%)	1.74	0.96–3.18	0.07

Asthma and oseltamivir treatment were found to be a protective factor.

## 4 DISCUSSION

This study focuses on adult patients admitted to the ICU for severe confirmed influenza infection. The objectives of the study were, first, to describe the severity of the disease, its potential complications, and evolution during ICU stay and, second, to analyze the impact of demographics, underlying conditions, influenza vaccination and treatment, bacterial coinfection, severity, and ICU management of influenza on ICU mortality and outcome.

The univariate analysis found risk factors of ICU mortality to be only patients over age 65 years and patients with a previous history of cancer and bacterial coinfections.

Underlying conditions such as diabetes mellitus, smoking, COPD, cystic fibrosis, coronary diseases, overweight, hypertension, renal insufficiency, history of cancer, immunosuppression status, pregnancy, and age over 65 years are considered as recognized risk factors for developing a severe influenza disease.^8^, ^9^ The literature is consistent and emphasizes that the presence of one of these factors is related with an increased risk for hospitalization.^8^, ^9^, ^10^

The characteristics of the population in Réunion Island is slightly different from those of mainland France and Western Europe. Diabetes mellitus is more frequent in Réunion Island's population with a 17.7% rate that is two times higher than in mainland France. Diabetes mellitus complications as renal insufficiency, and coronary diseases are of major concerns. Obesity is frequent as 35% of the entire population is overweight (17% in France).^11^ Smoking habits are recorded in 32% of the population (25% in France). Asthma is more frequent in children (19%) than in mainland France (11%).^12^ Previous studies showed identical results although with fewer number of patients. Kumar et al described in a study of the aboriginal population a similar high prevalence of underlying conditions versus the non‐aboriginal population living in Canada.^13^

In the present study, three factors increasing ICU mortality were found. Age >65 years was statistically related with mortality. This result is consistent with the literature. Only the Spanish outbreak in 1918 showed an abnormal percentage of deaths in young adults.^14^

History of cancer was the other factor related with fatality in the study and was recorded in 40 patients (11.5% of study population). No information on the type of cancer was available. Fifteen patients had ongoing chemotherapy or radiotherapy at ICU admission. Due to a small number of cases, immunosuppression drug therapy (16 patients) did not reach statistical significance, opposing recent data.^15^

Few studies focused on specific ICU populations of severe influenza, and some of them included a small patient cohort. Beumer et al analyzed 45 patients admitted in ICU for severe influenza.^16^ Considering the univariate analysis, history of diabetes mellitus and renal insufficiency were the two factors related with ICU death. The impact of age on mortality was however not analyzed in their study. Gilca et al analyzed the odds ratio for mortality in 62 ICU‐admitted patients compared to 259 hospitalized patients and found patients >60 years and immunosuppression treatment to be factors related to mortality.^17^

On the other hand, asthma was found as a protective factor. The mean age of asthmatics was 55.9 years of age and was not different from the overall study population. Twenty‐four of them were over 65 years of age (38%). In other studies, asthma was considered as a pejorative factor for hospitalization.^17^ COPD and asthma were frequent in our study population, but smoking habits and smoking‐related COPD were not found as independent factors for death.

The impact of therapy based on inhaled corticosteroids (ICS) widely used in asthma patients could be the reason for our finding. Recently, attention has been drawn on a potential protective effect of ICS in asthmatic patients with SARS COV‐2 infection,^18^ and a recent meta‐analysis confirmed the absence of increased risk for influenza and safety profile for asthmatics with regular use of ICS.^19^

Considering all the usual underlying conditions described in the study, the analysis of the literature confirmed that these factors are generally related with an increase for hospitalization in medical wards and secondary ICU admissions.^20^, ^21^ The specific impact on ICU's outcome is less evident and requires larger series.

Information about influenza vaccination was only available in 96 (27%) of patients. Among these patients, only 16 were vaccinated within the previous 12 months. Due to this small number, statistical analysis did not show any benefit. From a public health standpoint, influenza vaccination is therefore highly recommended in these populations.^8^, ^10^

The primary objective of this large study was to analyze patient's severity at the time of ICU admission and its evolution during the ICU stay.

We observed a crude mortality in the ICU of 25.1%. There are few studies analyzing the mortality in ICU‐admitted patients with severe influenza infection.

An old meta‐analysis found a wide range of crude mortality from 14% to 71%.^22^ More recently, Chao et al recorded deaths in 33/125 patients (26.4%), Abazieu et al in 20/69 patients (29%), and Beumer et al in 17/45 patients (38%).^16^, ^23^, ^24^ The results, based on a larger series, confirm the expected rate of deaths in severe patients admitted in ICU for influenza. Réunion's ICU's care is comparable to mainland France as all patients had access to MV with prone position if required, dialysis, and ECMO as needed.

The severity of patients at the time of admission in the ICU was described. The SAPS II score is a valid indicator of severity in ICU. A mean SPAS II score of 43.8 +/− 20.3 was considered severe and strongly related with ICU's poor outcome. In the univariate analysis, observations revealed a strong link with mortality when patients had a SAPS II score over 40 (*p* < 0.0001, RR 4.81). Another indicator was the need of invasive MV that reached 58% of patients. The majority of patients initially had respiratory failure as demonstrated by 81 (23.1%) of them requiring noninvasive MV. Only 65 (18.5%) patients received only oxygen. An important rate of ARDS cases (40.2%) was observed with ECMO requirement for 27 patients.

There is little specific information on this topic in the literature. In the cohort, Chen et al observed a moderate rate (18.9%) of MV requirement,^15^ and 24.6% of all patients had respiratory failure. In a French study, Abaziou et al reported 69 patients in ICU with a high ARDS percentage 50/69 (72.5%).^24^ The direct mortality during ICU stay was not indicated, but they observed a 24.6% mortality at 30 days after admission. In this study, the number of patients with ARDS was severe as 129 of them were intubated (96%), indicating that ARDS was considered as present only in the case of severe oxygenation impairment. ARDS grade III in 86/107 (80.4%) patients was recorded, and this is consistent with a high percentage of very severe cases and the related poor outcome as ARDS was a factor for mortality with a 4.31 RR, *p* < 0.0001. Kumar et al analyzed the ratio of PaO_2_ to FIO_2_ along days for 168 patients.^13^ This mean ratio was under 147 mmHg in Day 1 for 168 patients and 168 mmHg in Day 3 for 156 patients, indicating more moderate ARDS cases in their patients compared to our data. The major factor for mortality in their study was organ dysfunction in ICU.

ECMO is considered as the ultimate technique for refractory ARDS. Twenty‐seven patients required ECMO, and 21 (77%) died (*p* < 0.0001). The study of Abaziou et al focused on ECMO benefit for severe influenza.^24^ They used ECMO in 23 patients with a death rate of 39% (nine patients). The small number of patients with ECMO in each series may explain this difference. A learning curve for the technique may also be considered. A large series evaluating ECMO in ARDS did not find a significant benefit at 60 days.^25^

H1NI strains were the predominant virus with 43.7% of cases but were not correlated with mortality. The impact of the new swine origin influenza A (H1N1) strain on increase in hospitalization and death was recorded during the 2009 pandemic.^1^ No differences were seen in the cohort along years.

Oseltamivir was the unique anti‐influenza drug available in the study and was administrated at hospital or ICU admission in 229 (66.4%) of patients with a benefit in mortality reduction. The favorable impact of oseltamivir in influenza was previously described in the general population^26^ and in ICU patients.^17^, ^27^ However, early treatment is associated with a mortality reduction; therefore, administration is recommended within 2 days after symptom onset. Over 90% of patients were directly admitted to the ICU from the emergency rooms of the hospital. Oseltamivir was started in the ICU within the 48 h. The time between the first symptoms of influenza and the admission to the ICU was recorded for 327 patients. The mean duration of symptoms pending ICU admission was 4.9 +/− 4.2 days, including time prior to hospital admission. Despite this delay, a reduction in mortality related to oseltamivir use was observed. The efficacy of oseltamivir in the ICU has been fully demonstrated, but there are some studies denying this issue with no evidence of improvement for ICU patients receiving early or late oseltamivir therapy.^28^, ^29^ Nevertheless, based on the results, it is recommended to consider antiviral therapy with oseltamivir as soon as possible for all patients admitted to the ICU.

Bacterial coinfection is frequent in patients with influenza and was reported between 11% and 35%.^21^ The results confirm the high frequency of bacterial coinfections and were related to a mortality increase.^30^ Surprisingly, coinfection with either *S. aureus* or pneumococcus pneumoniae strains was not statistically correlated with mortality. Considering these results, it is advised to diagnose the presence of bacterial coinfections at admission in hospital and in the ICU to avoid delaying the introduction of effective antibiotic therapy.

As the study was of a retrospective nature, the limitations due to data collection are acknowledged. A lack of information is important for BMI and vaccination status. In life‐threatening situation, information on height and weight may not be systematically recorded. History of influenza vaccination is sometimes difficult to assess in ICU due to patient's condition and family questioning. For chronic diseases, compliance to daily therapy is impossible to assess. Patient's underlying conditions may be imprecise and may explain discrepancies in the results compared to literature. During hospitalization, the exact timing of starting oseltamivir was difficult to record, but the majority of patients were rapidly admitted to the ICU, and oseltamivir was early administered.

## 5 CONCLUSION

In summary, a large cohort of ICU‐admitted patients for severe influenza disease over 10 years was analyzed. The overall mortality was 25.1%. The predominant clinical presentation was a specific pulmonary involvement rapidly requiring MV and often evolving toward ARDS. Timely ICU admission for patients with severe influenza is recommended and as early as possible in the case of respiratory failure in order to enable effective intensive care.

Patient's underlying conditions are important to consider. Most of them are factors that influence hospital admission and secondary ICU admission and were not found to impact specific ICU mortality. In the study of ICU‐admitted patients presenting with severe influenza, patient age >65 years, history of cancer, and bacterial coinfections were identified as the three pejorative prognostic factors. Influenza vaccination remains important to promote in the community for elderly people and patients with comorbidities.

## AUTHOR CONTRIBUTIONS

Victor Verdier collected the data and wrote the manuscript. Fabrice Paganin analyzed the data and wrote the manuscript. Francois Lilienthal, Arnaud Desvergez, Arnaud Winer, and Virgile Gazaille wrote the manuscript. All the authors contributed equally.

## CONFLICT OF INTEREST STATEMENT

All the authors declare that they have no conflict of interest.

### PEER REVIEW

The peer review history for this article is available at https://www.webofscience.com/api/gateway/wos/peer-review/10.1111/irv.13168.

## ETHICS STATEMENT

We declare the study to the French Comite national de l'informatique et des libertés (CNIL) and received approval on May 15, 2018 (Number 2181973 V0). All data were kept anonymous. Not applicable for this section.

## Data Availability

The datasets used and/or analyzed during the current study are available from the corresponding author on reasonable request (data were collected on Excel files).
